# Ipilimumab- and Nivolumab-Induced Colitis Causing Severe Hypokalemia and QTc Prolongation

**DOI:** 10.1155/2019/7896749

**Published:** 2019-12-26

**Authors:** David Anson, Joseph Norton, Benjamin Chaucer, Saurabh Bansal

**Affiliations:** ^1^University of Illinois College of Medicine Peoria, Peoria, IL, USA; ^2^Department of Internal Medicine, University of Illinois College of Medicine Peoria, Peoria, IL, USA

## Abstract

Immune-mediated colitis is an uncommon but well-documented adverse event in patients receiving nivolumab or ipilimumab therapy. In this report, we present a 69-year-old man who developed severe hypokalemia and colitis with significant corrected Q-T segment (QTc) prolongation as a result of combination nivolumab-ipilimumab immunotherapy for clear cell renal cell carcinoma.

## 1. Introduction

We present the unique findings of severe hypokalemia and immune-mediated colitis from ipilimumab and nivolumab combination immunotherapy for clear cell renal cell carcinoma. The broad differential for severe diarrhea and electrolyte imbalance in a patient with malignancy and immunocompromised status receiving novel chemotherapeutic agents presents a significant diagnostic challenge.

## 2. Case Description

A 69-year-old man with a past medical history of metastatic renal cell carcinoma, diabetes mellitus type 2, and chronic kidney disease presented with a 2-month duration of frequent watery stools not relieved by metronidazole, atropine-diphenoxylate, or loperamide. He reported 10-12 loose, watery, brown, mucousy stools daily without gross blood or associated abdominal pain. The frequency and severity of diarrhea had progressively worsened over the last month. Combination immunomodulatory therapy of ipilimumab and nivolumab was started 3 months prior to arrival. He received his last therapy cycle 1 week prior to hospitalization. On admission, he presented with hypokalemia of 2.2 mmol/L, creatinine of 2.59 mg/dL, and orthostatic hypotension. Electrocardiogram (EKG) demonstrated QTc prolongation at 725 ms, normal anion gap metabolic acidosis with bicarbonate of 9 mmol/L. Abdominal radiography (KUB) showed gaseous distention of the small and large bowel and likely ileus. CT imaging was not conducted. Due to high risk of perforation in the acute setting, colonoscopy was not performed. Gentle fluid resuscitation, aggressive potassium repletion, and serial electrolyte monitoring were initiated. Stool studies for ova, parasites, *Clostridium difficile*, *Salmonella*, and bacterial enterotoxins were negative. Stool lactoferrin was positive. Adrenal insufficiency was suspected, but ruled out with normal morning cortisol and adrenocorticotropin stimulation testing. Thyroid hormone levels were normal. A diagnosis of immunotherapy-induced colitis was made. Treatment with intravenous methyl-prednisolone 60 mg daily for four days was initiated, and the patient's condition significantly improved. Diarrhea slowed down, electrolytes normalized, and EKG showed QTc improvement at 538 ms. After discussion with oncology, ipilimumab and nivolumab therapy was discontinued. He was discharged home with a four-week oral steroid taper regimen. The taper schedule was prednisone 50 mg for two weeks, followed by 30 mg for five days, then 20 mg for five days, and finally 10 mg for four days.

## 3. Discussion

This case illustrates the potential for severe electrolyte imbalance from immune-mediated colitis with the use of immunotherapeutic agents. Immune-mediated colitis is a well-documented adverse effect of immune checkpoint inhibitors (ICIs), with an incidence ranging from 1 to 25% depending on the ICI and whether the therapy is single agent or combination [1]. In addition, combined anti-CTLA4 and anti-PD-1 therapy significantly increases the frequency and severity of immune-mediated colitis (1). The National Institutes of Health and National Cancer Institute “Common Terminology Criteria for Adverse Events” (CTCAE) grading schema may be helpful for characterizing the severity of immune checkpoint inhibitor-induced colitis [2]. Based on the CTCAE grading scale, our patient would be categorized as grade 2 colitis, due to mucus in the stool, and grade 4 diarrhea, due to severe electrolyte derangement with profound widening of the Q-R-S complex evidenced by QTc prolongation (see Table 1).

Previous case reports have described severe colitis secondary to combined ipilimumab and nivolumab therapy refractory to high-dose steroid therapy [3]. While our patient demonstrated a rapid improvement after initiation of steroids, up to 40% of cases may require initiation of infliximab as a second-line therapy (4). Like the majority of cases reported in the literature, our patient developed severe diarrhea and worsening colitis shortly after receiving his third cycle of combination therapy. Risk factors for the development of ICI-induced colitis include history of autoimmune diseases, nonsteroidal anti-inflammatory drug use, and baseline gut microbiome composition (1). Other documented, serious adverse effects include hepatitis, adrenal insufficiency, hypothyroidism, pancreatic dysfunction, and myocarditis [4].

While the occurrence and treatment of these conditions have been documented, the exact mechanism by which these events occur is not well understood. In nivolumab, current hypotheses suggest a role of T-cell activation by the antibody which results in an enhanced immune response and that this effect may be compounded in the presence of ipilimumab [5]. Interestingly, nivolumab-induced colitis exhibits endoscopic features similar to that of ulcerative colitis (UC) and may have a therapeutic response to agents such as mesalazine typically used in UC and other inflammatory bowel disease [6]. Recently, clinicians have also documented faster resolution of symptoms with high-dose steroid therapy as opposed to the traditional guidance of lower dose treatment, which may prolong hospitalization [7]. Treatment strategy of combination ipilimumab-nivolumab colitis consists of cessation of immunomodulatory therapy, initiation of steroid therapy, and supportive treatment. In steroid-refractory cases, infliximab or vedolizumab therapy may be utilized as a second-line regimen. In addition, fecal microbiota transplantation may be considered in patients in which steroid and anti-TNF therapies have proven unsuccessful (1). Careful surveillance of serum potassium during steroid therapy is recommended, as corticosteroids may cause hypokalemia. This is especially pertinent during ongoing gastrointestinal losses when there is minimal improvement after several days of steroid administration.

## 4. Conclusion

The broad range of possible presentations in immunotherapy-induced dysfunction may prove a particularly challenging entity to identify and treat appropriately. Unfortunately, immunotherapeutic side effect profiles are not well known within the medical community. As the use of immunotherapy becomes more prevalent, prompt diagnosis and cessation of the offending agent, initiation of treatment with steroids, and supportive treatment in immunotherapy-induced colitis are vital to preventing unfavorable outcomes.

## Figures and Tables

**Table 1:** Common Terminology Criteria for Adverse Events grading scale in immune checkpoint inhibitor-induced colitis and diarrhea.

	Colitis	Diarrhea
Grade 1	Asymptomatic	Increase of <4 stools/day
Grade 2	Abdominal pain, mucus, blood in stool	Increase of 4-6 stools/day
Grade 3	Severe pain, fever, peritoneal signs	Increase of ≥7 stools/day
Grade 4	Life-threatening consequences (perforation, ischemia, necrosis, bleeding, toxic megacolon)	Life-threatening consequences such as hemodynamic collapse
Grade 5	Death	Death
